# Co-administration of a *Rhododendron tomentosum* extract does not affect mercury tissue concentrations and excretion rate in methylmercury-treated adult male rats

**DOI:** 10.1186/s13104-019-4409-7

**Published:** 2019-07-01

**Authors:** Guillaume Pelletier, Yong-Lai Feng, Karen Leingartner, Paleah Black

**Affiliations:** 1 Hazard Identification Division, Environmental Health Science and Research Bureau, Health Canada, Environmental Health Centre, 50 Colombine Driveway, P.L. 0803B, Tunney’s Pasture, Ottawa, ON K1A 0K9 Canada; 2 Exposure and Biomonitoring Division, Environmental Health Science and Research Bureau, Health Canada, Ottawa, ON Canada; 3 Department of Biology, University of Ottawa, Ottawa, ON Canada

**Keywords:** Rat, Co-exposure, Methylmercury, Labrador Tea, *Rhododendron tomentosum*, Antibiotics, Gut microbiota

## Abstract

**Objectives:**

Consumption of fish/seafood is clearly linked to higher mercury levels in human tissue samples. However, correlations between methylmercury (MeHg) intakes calculated from dietary surveys and mercury body burdens are usually weak and can vary across populations. Different factors may affect MeHg absorption, distribution, metabolism and excretion, including co-exposures to phytochemicals and antibiotics, which were shown to affect mercury body burdens in rodents. Based on the observation that rat pups developmentally exposed to MeHg and a *Rhododendron tomentosum* extract (Labrador Tea) presented significantly higher blood mercury levels at weaning compared to pups exposed to MeHg alone, the modulation of MeHg toxicokinetics by Labrador Tea was further investigated in adult rats.

**Results:**

Total mercury levels were quantified in the blood, liver, kidney and feces of adult male rats exposed to MeHg (1.2 mg/kg bodyweight/day, for 3 weeks) administered either alone or in combination with Labrador Tea (100 mg/kg bodyweight/day) or with an antibiotics cocktail (to inhibit MeHg demethylation by gut bacteria). While the reduced fecal excretion and higher blood mercury levels expected from antibiotics-treated rats were observed, mercury levels in samples from Labrador Tea-treated rats were not significantly different from those measured in samples from rats exposed to MeHg alone.

**Electronic supplementary material:**

The online version of this article (10.1186/s13104-019-4409-7) contains supplementary material, which is available to authorized users.

## Introduction

Although epidemiology studies clearly link increased mercury levels in human tissue samples to fish and seafood consumption, correlation coefficients between estimated MeHg dietary intakes and body burdens are usually weak, ranging from 0.3 to 0.4 [1, 2]. These relatively weak correlations have been attributed to mercury quantification issues, dietary survey imprecisions, recall biases [2, 3], impaired liver functions [4, 5], genetic polymorphisms [6, 7], cooking methods [8, 9], and dietary interactions [6, 8–12]. A better understanding of the factors affecting MeHg absorption, distribution, metabolism and excretion may contribute to a refinement of risk assessments, regulatory guidelines and mitigation strategies.

Methylmercury present in food (or excreted in bile) is almost entirely absorbed (or reabsorbed) in the gut [13]. However, a fraction of MeHg escapes enterohepatic cycling through demethylation in the gut lumen, which results in the production of poorly absorbed inorganic mercury [14]. Gut bacteria play an important role in this process, as illustrated by the slower elimination of MeHg observed in germ-free or antibiotic-treated rodents [15–17]. The gut microbiome can also be affected by diet [18], as commonly consumed phytochemicals can present bactericidal or bacteriostatic properties in vitro [19, 20]. Interestingly, consumption of black tea has been shown to affect the human gut microflora [21], and volunteers who ate fish and drank tea presented higher blood mercury concentrations than volunteers who only ate fish in a controlled human exposure study [22].

We previously reported that male and female rat pups developmentally exposed to MeHg and Labrador Tea (an antioxidant-rich *Rhododendron tomentosum* decoction consumed in Canadian Arctic communities) presented significantly higher blood mercury levels at weaning compared to pups exposed to the same MeHg dose administered alone [23]. In this follow-up study, adult male rats were exposed to the same daily dose of Labrador Tea or to an antibiotics cocktail (a positive control for gut bacteria inhibition) for 4 weeks (days 1 to 28), with or without concomitant daily exposure to MeHg for 3 weeks (days 8 to 28). At the end of the exposure period, total mercury concentrations in the blood, liver, kidney and feces of MeHg-treated rats co-exposed to Labrador Tea or antibiotics were compared to those measured in rats exposed to MeHg alone.

## Main text

### Methods

#### Materials

*Rhododendron tomentosum* ssp. *subarcticum* collection and characterization of the ethanolic extract were described by Black et al. [24]. The same freeze-dried extract (Labrador Tea) conserved under nitrogen gas in amber vials at − 20 °C was used to dose rat pups in a previous study [23] and adult rats in this study. The corn oil (Mazola brand, ACH foods, Memphis, TN, USA) and cookies (Teddy Graham, Nabisco, Toronto, ON, Canada) were purchased from a local grocery store. Unless otherwise stated, reagents were purchased from Sigma-Aldrich (Oakville, ON, Canada).

#### Animal treatment

Experimental procedures were approved by Health Canada’s Institutional Animal Care Committee. Sprague–Dawley male rats (7–8 weeks old) were purchased from Charles River Laboratories (St. Constant, QC, Canada) and housed individually in Health Guard^R^ cages (Research Equipment Co., Bryant, TX, USA). They were allowed to acclimatize to the facility (22 ± 1 °C, 50 ± 10% humidity and 12-h [7:00 a.m. to 7:00 p.m.] light cycle) for at least 10 days. Teklad 2014 rodent diet (Harlan Laboratories, Indianapolis, IN, USA) and water were provided ad libitum. Rats were also trained to accept a cookie used for dosing.

Seven rats were randomly assigned to each of the following treatment groups: control, Labrador Tea (100 mg/kg bw/day), antibiotics (neomycin [2 g/L], streptomycin [2 g/L] and penicillin [2 g/L], in drinking water), MeHg (1.2 mg/kg bw/day), MeHg + Labrador Tea and MeHg + antibiotics. Rats were weighed daily and water bottles changed twice a week. Rats were exposed to Labrador Tea and antibiotics from exposure day 1 to 28 and to MeHg from exposure day 8 to 28. Weight-adjusted volumes of Labrador Tea dissolved in 100% ethanol were applied to cookies that were allowed to dry overnight in a fume hood. MeHg dissolved in corn oil was added to cookies (or to cookies containing Labrador Tea). To ensure uniform caloric intake, all rats received a sweet cookie (exposure day 1 to 7) or a sweet cookie laced with a weight-adjusted volume of corn oil (1.0 mL/kg bodyweight, exposure day 8 to 28).

Feces samples were collected on the 28th day of exposure and rats were sacrificed under isoflurane anaesthesia on the following morning. Blood was drawn from the aorta, organs were inspected and the liver and kidneys were weighed. Hematological parameters and enzymatic activities in liver S9 extracts were measured as described by Poon et al. [25, 26]. Serum IgE, IgG, IgA and IgM levels were measured using ELISA Test Kits (GenWay Biotech Inc, San Diego, CA, USA) according to the manufacturer’s instructions.

#### Mercury quantification in rat tissues

Total mercury measurements in blood and lyophilised kidney, liver and feces were performed by Prairie Diagnostic Services Inc. (Saskatoon, SK, Canada). Samples were digested in nitric acid in a Microwave Accelerated Reaction System (MARS-5, CEM, Matthews, NC, USA) and total mercury was quantified by inductively-coupled plasma mass spectrometry (ICAP Q ICP-MS, Thermo Scientific, Waltham, MA, USA) as previously described [27, 28].

#### Statistical analysis

All raw data are included in the Additional file 1. Datasets satisfying normality and homoscedasticity assumptions (Shapiro–Wilk’s test and Brown–Forsythe’s test) were analysed by one-way ANOVA followed by Dunnett post hoc test, using SigmaPlot 11.2 (Systat Software Inc., Chicago, IL, USA). Otherwise, datasets were log-transformed and analysed using Kruskal–Wallis ANOVA followed by Dunnett post hoc test on ranks if they still failed to satisfy normality and homoscedasticity assumptions. Mercury tissue concentrations in Labrador Tea or antibiotics-treated rats were compared to the control or MeHg treatment group, as appropriate.

### Results

None of the treatment significantly affected male rat weight gains (Table 1) or resulted in overt sign of toxicity over the dosing period. Beside kidney cysts observed in two control rats and in one rat from the MeHg + Labrador Tea treatment group, no other gross organ abnormality was observed at necropsy. Although the overall kidney cyst rate (3/42 rats or 7%) was high compared to spontaneous kidney cysts reported in 2-year old male rats from five different species (5–12%) [29], these cysts were not considered treatment-related as they were observed mainly in control rats. Lower relative liver weights accompanied by increased BROD, EROD and PROD activities were observed in the antibiotics treatment group. Both absolute and relative liver weights were significantly lower in the MeHg + antibiotics treatment group, but only BROD activity was significantly increased (Table 1).

**Table 1:** Effects of treatments on rat bodyweight gains, organ weights and liver enzymatic activities

	Control	Labrador Tea	Antibiotics	MeHg	MeHg + Labrador Tea	MeHg + antibiotics
Body weight gain (g)	78.0 ± 15.6	84.4 ± 8.0	82.9 ± 17.1	75.3 ± 22.1	75.7 ± 20.8	76.8 ± 21.2
Kidney weight, absolute (g)	2.20 ± 0.25	2.25 ± 0.24	2.24 ± 0.25	2.35 ± 0.22	2.41 ± 0.13	2.25 ± 0.26
Kidney weight, relative (%)	0.609 ± 0.029	0.606 ± 0.028	0.613 ± 0.029	0.657 ± 0.042	0.659 ± 0.055	0.615 ± 0.050
Liver weight, absolute (g)	12.64 ± 2.42	12.98 ± 0.88	10.60 ± 1.37	11.78 ± 1.06	12.44 ± 1.88	10.32 ± 1.34*
Liver weight, relative (%)	3.50 ± 0.17	3.50 ± 0.16	2.89 ± 0.17*	3.29 ± 0.19	3.37 ± 0.23	2.81 ± 0.16*
Liver enzymatic activities						
BROD (pmol/min/mg prot)	42.7 ± 18.7	88.6 ± 26.3	105.5 ± 49.1*	62.2 ± 23.4	84.0 ± 41.4	103.2 ± 36.3*
EROD (pmol/min/mg prot)	54.1 ± 24.4	88.4 ± 22.5	93.4 ± 37.6*	28.0 ± 18.6	63.8 ± 28.4	69.4 ± 21.7
PROD (pmol/min/mg prot)	17.3 ± 7.0	30.0 ± 8.8	33.5 ± 13.1*	16.6 ± 6.9	19.5 ± 10.0	22.1 ± 8.8
GGT (mU/g prot)	93.7 ± 51.0	113.2 ± 34.0	146.1 ± 30.1	102.9 ± 76.9	100.1 ± 32.5	79.8 ± 33.2

Mean ± SD of seven animals per treatment group

EROD: ethoxyresorufin-*O*-deethylase; BROD: benzyloxyresorufin-*O*-dealkylase; PROD: pentoxyresorufin-*O*-dealkylase; GGT: gamma glutamyl transferase

* Significantly different from control group values (P < 0.05) as assessed by one-way ANOVA followed by Dunnett post hoc test

Co-exposures to Labrador Tea and antibiotics did not significantly interfere with MeHg’s effects on the hematological parameters assessed. Mean corpuscular hemoglobin concentration (MCHC) was significantly decreased following exposure to antibiotics and in all treatment groups exposed to MeHg (Table 2). The expected increases in serum IgE levels [30, 31] were observed in all MeHg-treated rats, while a modest increase in IgG concentration of dubious biological relevance was observed in the MeHg + Labrador Tea treatment group (Table 2).

**Table 2:** Effects of treatments on rat hematological parameters

	Control	Labrador Tea	Antibiotics	MeHg	MeHg + Labrador Tea	MeHg + antibiotics
RBC (× 10^6^ cells/µL)	7.54 ± 0.46	7.59 ± 0.15	7.93 ± 0.32	7.83 ± 0.32	7.86 ± 0.29	7.75 ± 0.37
HGB (g/dL)	14.36 ± 0.49	14.11 ± 0.34	14.37 ± 0.43	14.39 ± 0.29	14.37 ± 0.61	14.33 ± 0.54
HCT (%)	42.31 ± 1.95	42.01 ± 1.50	43.50 ± 1.08	43.84 ± 1.35	43.67 ± 1.92	43.57 ± 1.82
MCV (fL)	56.20 ± 1.10	55.36 ± 1.18	54.95 ± 1.42	56.03 ± 1.77	55.53 ± 1.69	56.24 ± 2.00
MCH (pg)	19.07 ± 0.70	18.59 ± 0.31	18.18 ± 0.43	18.4 ± 0.59	18.28 ± 0.48	18.46 ± 0.59
MCHC (g/dL)	33.94 ± 0.72	33.59 ± 0.54	33.03 ± 0.64*	32.83 ± 0.63*	32.88 ± 0.58*	32.86 ± 0.32*
PLT (× 10^3^ cells/µL)	867 ± 138	1010 ± 68*	921 ± 84	977 ± 45	969 ± 62	875 ± 159
MPV (fL)	6.03 ± 1.29	6.04 ± 1.11	6.08 ± 1.34	6.20 ± 1.08	6.40 ± 1.38	6.20 ± 1.37
WBC (× 10^3^ cells/µL)	4.25 ± 1.57	4.62 ± 0.39	3.86 ± 1.50	5.79 ± 1.72	5.13 ± 1.4	4.24 ± 1.10
LYM (× 10^3^ cells/µL)	3.21 ± 1.63	3.79 ± 0.37	3.26 ± 1.47	4.35 ± 1.53	3.92 ± 1.25	3.32 ± 0.75
NEUT (× 10^3^ cells/µL)	0.57 ± 0.46	0.41 ± 0.25	0.30 ± 0.19	0.78 ± 0.90	0.5 ± 0.36	0.23 ± 0.16
MONO (× 10^3^ cells/µL)	0.23 ± 0.22	0.22 ± 0.31	0.12 ± 0.12	0.32 ± 0.37	0.36 ± 0.39	0.29 ± 0.29
EOS (× 10^3^ cells/µL)	0.041 ± 0.018	0.040 ± 0.008	0.040 ± 0.030	0.049 ± 0.020	0.073 ± 0.077	0.053 ± 0.027
LUC (× 10^3^ cells/µL)	0.17 ± 0.14	0.14 ± 0.17	0.12 ± 0.12	0.25 ± 0.32	0.23 ± 0.25	0.30 ± 0.34
BASO (× 10^3^ cells/µL)	0.03 ± 0.01	0.02 ± 0.00	0.03 ± 0.01	0.04 ± 0.02	0.03 ± 0.01	0.03 ± 0.02
LYM (%)	73.26 ± 13.50	82.01 ± 5.09	82.28 ± 7.63	75.09 ± 12.07	76.03 ± 5.22	79.13 ± 7.07
NEUT (%)	14.56 ± 12.89	9.06 ± 5.73	8.33 ± 5.30	14.09 ± 14.28	10.42 ± 7.35	6.16 ± 4.49
MONO (%)	6.11 ± 6.35	4.64 ± 6.40	3.65 ± 4.75	5.24 ± 5.68	7.05 ± 7.82	6.31 ± 5.33
EOS (%)	1.04 ± 0.46	0.90 ± 0.21	1.08 ± 0.73	0.91 ± 0.51	1.58 ± 1.83	1.34 ± 0.57
LUC (%)	4.36 ± 3.93	2.96 ± 3.35	3.73 ± 4.81	4.07 ± 5.04	4.33 ± 4.4	6.21 ± 6.14
BASO (%)	0.66 ± 0.32	0.43 ± 0.10	0.93 ± 0.79	0.61 ± 0.21	0.63 ± 0.18	0.89 ± 0.50
IgG (mg/mL)	2.57 ± 0.93	3.42 ± 0.93	2.77 ± 0.97	3.48 ± 1.71	5.48 ± 2.21^#^	2.89 ± 1.62
IgM (µg/mL)	108.2 ± 38.9	94.8 ± 21.9	84.1 ± 18.7	218.4 ± 303.5	107 ± 18.3	85.9 ± 29.8
IgA (µg/mL)	22.7 ± 3.4	24.7 ± 2.6	24.9 ± 12.0	18.7 ± 2.7	38.2 ± 11.7	22.4 ± 3.2
IgE (ng/mL)	9.2 ± 9.1	14.5 ± 26.1	6.3 ± 6.1	240.3 ± 263.6*	410.1 ± 266.7*	115.9 ± 199.3*

Mean ± SD of 6–7 animals per treatment group

RBC: red blood cells; HGB: hemoglobin; HCT: hematocrit; MCV: mean corpuscular volume; MCH: mean corpuscular hemoglobin; MCHC: mean corpuscular hemoglobin concentration; PLT: platelet; MPV: mean platelet volume; WBC: white blood cells; LYM: lymphocytes; NEUT: neutrophils; MONO, monocytes; EOS: eosinophils; LUC: large unstained cells; BASO: basophils

* Significantly different from control group values (P < 0.05) as assessed by one-way ANOVA followed by Dunnett post hoc test

^#^Significantly different from control group values (P < 0.05) as assessed by Kruskal–Wallis ANOVA followed by Dunnett post hoc test on ranks

Rats from the antibiotics treatment group presented significantly higher blood and kidney mercury levels than those observed in the control group (Table 3). In MeHg-treated rats, co-exposure to antibiotics resulted in lower feces and higher blood mercury levels. Contrastingly, mercury levels measured in the Labrador Tea and MeHg + Labrador Tea treatment groups were undistinguishable from those observed in the control or MeHg treatment groups, respectively (Table 3).

**Table 3:** Total mercury concentrations measured in rat blood and lyophilized liver, kidney and feces

	Control	Labrador Tea	Antibiotics	MeHg	MeHg + Labrador Tea	MeHg + antibiotics
Blood (ppm)	0.0231 ± 0.0035	0.0262 ± 0.0019	0.0441 ± 0.0080*	76.4 ± 6.5	74.9 ± 10.5	98.1 ± 18.0*
Liver (ppm)	0.0235 ± 0.0028	0.0238 ± 0.0122	0.0348 ± 0.0068	53.1 ± 5.0	50.7 ± 13.2	45.5 ± 7.0
Kidney (ppm)	0.189 ± 0.027	0.202 ± 0.027	0.353 ± 0.055*	343.1 ± 24.5	334.8 ± 57.7	325.3 ± 48.9
Feces (ppm)	0.0208 ± 0.0090	0.0177 ± 0.0014	0.018 ± 0.010	16.4 ± 4.3	15.7 ± 3.5	5.4 ± 0.7*

Mean ± SD of 6–7 animals per treatment group

* Significantly different from control group or MeHg treatment group values (P < 0.05) as assessed by one-way ANOVA followed by Dunnett post hoc test

### Discussion

Information on the modulation of MeHg toxicokinetics by dietary phytochemicals in human is scarce, especially for Arctic populations [32, 33]. In a controlled exposure study, tea consumption was associated with higher blood mercury levels in volunteers who ate fish [22], while higher maternal blood mercury levels were associated with herbal tea consumption in a large British birth cohort study [1]. In rodents, similarly to our previous work on perinatal co-exposure to MeHg and Labrador Tea [23], other investigators reported that co-administration of green tea extract to fish-fed rats led to higher blood mercury levels [34], and that mice co-exposed to green tea extract and MeHg presented higher muscle mercury levels than mice exposed to MeHg alone [35]. Together, these investigations suggest that dietary phytochemicals may interfere with MeHg absorption, distribution, metabolism and excretion.

In the current study, MeHg, Labrador Tea and antibiotics administered either alone or in combination, did not affect rat bodyweight gains (Table 1) or cause overt toxicity over the exposure period. They also had limited effects on the hematological parameters assessed (Table 2). Hence, indirect effects resulting from severe toxicity are unlikely to have interfered with MeHg absorption, distribution, metabolism and excretion. Gamma glutamyl transferase (GGT) activity, which is involved in the biliary metabolism of MeHg-glutathione conjugate to the more easily reabsorbed MeHg-cysteine [13], was not significantly affected by any of the treatment groups (Table 1). Despite in vitro data suggesting that Labrador Tea may affect cytochrome p450 activities [36], no statistically significant perturbation of liver enzymatic activities (Table 1) was observed following exposure to Labrador Tea at a concentration previously shown to affect oxidative stress biomarkers and blood MeHg levels in developing rat pups [23].

As expected, co-administration of MeHg and antibiotics resulted in lower fecal excretion rates and higher blood mercury levels compared to rats exposed to MeHg alone (Table 3). In rats that were not exposed to MeHg, the antibiotics treatment group also presented higher blood mercury levels compared to the control group. At background mercury exposure levels, MeHg represents a comparatively smaller fraction of the total mercury measured in rats [23]. This may explain why fecal mercury excretion (which is influenced by MeHg demethylation [15–17]) was not significantly affected, while kidneys (which preferentially accumulate inorganic mercury [37]) presented significantly higher mercury levels in the antibiotics treatment group.

Contrary to our previous rat perinatal MeHg co-exposure study where Labrador Tea significantly affected blood mercury levels in both male and female pups [23], co-administration of Labrador Tea had no effect on mercury body burden or excretion in adult male rats (Table 3). This observation may reflect genuine differences between juvenile and adult rats in the absorption, distribution, metabolism and excretion of MeHg. Alternatively, rat pups are acutely sensitive to MeHg toxicity and high mortality rates were observed in developmentally exposed pups [23]. The mitigation of MeHg-induced oxidative stress and excitotoxicity by Labrador Tea observed in this previous study may therefore have resulted in the improved survival of rats pups presenting higher mercury body burdens, compared to pups exposed to MeHg alone [23].

Although Labrador Tea consumption is limited to small Northern/Arctic populations, the most abundant phytochemicals identified in *R. tomentosum* extract, namely quercetin, cachetins, chlorogenic acid and caffeic acid [24], are commonly found in various other food sources and can be purchased as dietary supplements. While we were able to clearly observe the effects of antibiotics treatment, we failed to detect any significant effect of Labrador Tea co-exposure on mercury fecal excretion and body burden in adult male rats. This observation is at odds with our previous Labrador Tea co-exposure study in rats pups [23] and with other MeHg and tea co-exposure studies in rodents [34, 35] and humans [22]. However, in light of the well-known literature bias towards positive results [38], it is nevertheless important to report such negative findings.

## Limitations

Although the lower fecal excretion rates and higher blood mercury levels observed in antibiotics-treated rats were attributed to the inhibition of MeHg demethylation by gut bacteria, other mechanisms such as perturbation of enzymatic activities in the liver (Table 1) or in other tissues, and alteration of gut barrier permeability [39] may also have contributed to these observations. Likewise, phytochemicals may also affect MeHg toxicokinetics through similar mechanisms, in addition to modulation of gut microbiota [19–21] and MeHg chelation [9, 11, 40]. Generalization of our conclusions to other teas, fruits and vegetables should be avoided, as phytochemicals absent from Labrador Tea but present in other herbal decoctions, and dietary fibers in fruits and vegetables may affect MeHg absorption, distribution, metabolism and excretion [11, 41]. Finally, we cannot rule out that a different choice of exposure and experimental protocols may have allowed the observation of subtler effects.

## Additional file

**Additional file 1.** Additional file contains all the raw data on rat body weight gains, organ weights, hematology, antibody isotyping, liver functions and mercury quantification presented in this manuscript.

## Data Availability

The raw data for this manuscript are included in Additional file 1.

## Abbreviations

ANOVA: analysis of variance
BASO: basophils
BROD: benzyloxyresorufin-*O*-dealkylase
EOS: eosinophils
EROD: ethoxyresorufin-*O*-deethylase
GGT: gamma glutamyl transferase
HCT: hematocrit
HGB: hemoglobin
ICP-MS: inductively-coupled plasma mass spectrometry
IgA: immunoglobulin A
IgE: immunoglobulin E
IgG: immunoglobulin G
IgM: immunoglobulin M
LUC: large unstained cells
LYM: lymphocytes
MCH: mean corpuscular hemoglobin
MCHC: mean corpuscular hemoglobin concentration
MCV: mean corpuscular volume
MeHg: methylmercury
MONO: monocytes
MPV: mean platelet volume
NEUT: neutrophils
PLT: platelet
PROD: pentoxyresorufin-*O*-dealkylase
RBC: red blood cells
SD: standard deviation
WBC: white blood cells
